# Low levels of the second messenger c-di-GMP enhance tolerance and resistance to meropenem in *Pseudomonas aeruginosa*

**DOI:** 10.3389/fcimb.2026.1775945

**Published:** 2026-03-09

**Authors:** Tarcisio Brignoli, Silvia Ferrara, Edoardo Labrini, Giovanni Bertoni

**Affiliations:** 1 Department of Biosciences, Università Degli Studi di Milano, Milan, Italy; 2 National Research Council, Institute of Biophysics, Milan, Italy

**Keywords:** antibiotic resistance, antibiotic tolerance, carbapenems, carbon catabolite repression, c-di-GMP, Hfq, meropenem, *Pseudomonas aeruginosa*

## Abstract

**Background:**

The carbapenem antibiotic meropenem is often used to treat life-threatening infections caused by *Pseudomonas aeruginosa*. Previous studies have shown that the susceptibility of *P. aeruginosa* to carbapenems is differentially regulated by the RNA chaperone Hfq, depending on the availability of preferred or less preferred carbon sources, a mechanism known as carbon catabolite repression (CCR). In this regulation, Hfq plays a CCR-conditioned repressive role on outer membrane porins that act as entry ports for carbapenems. In this study, we investigated whether meropenem response is modulated by the second messenger c-di-GMP, which is known to regulate several bacterial functions.

**Methods:**

We used *P. aeruginosa* strains with high or low c-di-GMP levels and their Hfq-deficient derivatives to assess the role of c-di-GMP in modulating meropenem susceptibility and tolerance.

**Results:**

We show that low intracellular c−di−GMP levels increase meropenem resistance and tolerance at sub−inhibitory concentrations, whereas high c−di−GMP diminishes both traits. Importantly, c−di−GMP status shapes the entire response trajectory, from exponential growth to the stationary phase. Furthermore, we show that c-di-GMP modulates meropenem response through mechanism(s) independent of Hfq-mediated porin repression and exerts a dominant effect over CCR-driven regulation.

**Conclusion:**

This study supports the notion that *P. aeruginosa* meropenem susceptibility and tolerance are modulated by intracellular c-di-GMP concentrations, with low c-di-GMP levels promoting higher fitness. Our findings indicate that c-di-GMP exerts its regulatory effect through mechanisms distinct from Hfq-mediated porin control, underscoring the existence of parallel regulatory pathways that shape antibiotic response.

## 1 Introduction

*Pseudomonas aeruginosa*, a prominent opportunistic pathogen responsible for severe hospital-acquired infections (Qin et al., 2022; Tuon et al., 2022), combines intrinsic antibiotic tolerance with adaptive resistance mechanisms driven by mutational changes and horizontal acquisition of resistance genes (Botelho et al., 2019). Carbapenems, a class of β-lactam antibiotics, are frontline agents against multidrug-resistant *P. aeruginosa* (Papp-Wallace et al., 2011; Glen and Lamont, 2021). They block peptidoglycan synthesis by targeting penicillin-binding proteins (PBPs), weakening the cell wall and inducing osmotic lysis. Consequently, rapidly dividing planktonic cells are more vulnerable to carbapenem killing than biofilm-associated cells, particularly those in nutrient- and oxygen-limited biofilm cores. In *P. aeruginosa*, carbapenems enter the periplasm via the outer membrane porins OprD and OpdP, which normally facilitate the uptake of basic amino acids and related nutrients (Tamber and Hancock, 2006; Eren et al., 2012; Glen and Lamont, 2021; Amisano et al., 2025).

*P. aeruginosa* rapidly evolves resistance under antibiotic pressure through multiple adaptive mechanisms (Pelegrin et al., 2021; Zhao et al., 2024). Adaptive carbapenem resistance in *P. aeruginosa* primarily arises through mutations and horizontal gene transfer that impair OprD-mediated uptake, upregulate efflux pumps, and promote β-lactamase-driven drug inactivation (Hassuna et al., 2020; Glen and Lamont, 2021; Fuhs et al., 2024). Beyond these genetic routes, carbapenem resistance is metabolically regulated by carbon catabolite repression (CCR), which modulates the expression of the porins OprD and OpdP through translation repression mediated by the RNA chaperone protein Hfq (Pusic et al., 2018; Sonnleitner et al., 2020; Sonnleitner, 2025). Repression of OprD involves Hfq-dependent small RNAs, while translation of OpdP is blocked by Hfq in concert with the protein Crc. CCR is fine-tuned by the small RNA CrcZ, whose expression depends on the carbon source. CrcZ antagonizes Hfq by binding and sequestering it, relieving repression under conditions where preferred carbon sources are absent. Through this dynamic interplay, CCR integrates metabolic status with outer membrane permeability, linking nutrient availability to carbapenem uptake and shaping antibiotic susceptibility and tolerance.

Coupling of metabolic status with antibiotic response reflects a multilayered regulatory network that transduces environmental cues into physiological adaptations influencing antibiotic uptake and survival. Beyond CCR, second messenger systems like c-di-GMP act as central hubs linking environmental signals to physiological adaptation, ensuring bacterial resilience under stress. C-di-GMP signaling regulates bacterial functions at multiple levels, highly integrated with other regulatory systems (Liu et al., 2024). C-di-GMP exerts its regulatory role by binding to multiple effector molecules, like proteins or RNA riboswitches, influencing their activity. As a result, c-di-GMP modulates gene transcription, mRNA translation, and protein function. C-di-GMP regulates several bacterial functions, but it is mostly regarded as a key regulator of the lifestyle transition from planktonic to sessile community biofilm (Valentini and Filloux, 2016). High c-di-GMP levels promote biofilm formation, while planktonic cells are associated with low c-di-GMP levels. The transition from planktonic growth to biofilm lifestyle is determined by drastic physiological changes, which include production of extracellular matrix and unique metabolic patterns compared to planktonic counterparts (Vital-Lopez et al., 2015; Malviya et al., 2023). Usually, bacterial biofilms are regarded as more resistant to antibiotics due to i) poor penetration of the antibiotics through the extracellular matrix and ii) metabolic heterogeneity of the bacterial community (Cao et al., 2015; Soares et al., 2020; Luo et al., 2021). Despite extensive research on biofilm-associated resistance, the role of c-di-GMP signaling in modulating antibiotic response remains poorly understood.

This study investigates how c-di-GMP signaling influences *P. aeruginosa* susceptibility and tolerance to meropenem, a key carbapenem used in clinical therapy (Hassuna et al., 2020; Fuhs et al., 2024). *P. aeruginosa* strains engineered for distinct c-di-GMP levels provided a framework to separate biofilm-related contributions from the intrinsic role of c-di-GMP signaling in shaping meropenem response. Our results show that low intracellular c−di−GMP levels increase meropenem resistance and tolerance at sub−inhibitory concentrations, whereas high c−di−GMP diminishes both traits. Importantly, c−di−GMP status shapes the entire response trajectory, from exponential growth to the stationary phase. Furthermore, we show that c-di-GMP modulates meropenem response through mechanism(s) independent of Hfq-mediated porin repression and exerts a dominant effect over CCR-driven regulation.

## 2 Materials and methods

### 2.1 Bacterial strains, plasmids, and culture conditions

Bacterial strains and plasmids used are listed in **Supplementary Tables 1A, B**, respectively. *P. aeruginosa* strains were grown at 37 °C in Luria-Bertani broth, unless specified differently. Experiments with the strains PAO1 Δ*pp*, PAO1 Δ*pp yfiN*^ind,^and PAO1 Δ*pp* PA2133^ind^ were conducted with LB media supplemented with 0.2% arabinose.

### 2.2 Mutant strain generation

PAO1 Δ*pp*, PAO1 Δ*pp yfiN*^ind^, PAO1 Δ*pp* PA2133^ind^ knock-out mutants in the *hfq* gene were obtained by allelic exchange using the pSEVApa14-Δ*hfq* plasmid as previously reported (Carloni et al., 2017). The pSEVAPA14-Δ*hfq* plasmid was transferred from *E. coli* CC118 λ*pir* to the receiver *P. aeruginosa* strains (PAO1 Δ*pp*, PAO1 Δ*pp yfiN*^ind^, or PAO1 Δ*pp* PA2133^ind^) through conjugative triparental mating, with the assistance of the helper *E. coli* strain HB101(pRK600). *P. aeruginosa* clones were selected on M9-citrate with 60 µg/mL gentamicin; integration of the plasmid was verified by PCR with the primers 1, 4, and 2, 4. The plasmid pSW-1 was transferred from *E. coli* DH5α to the *P. aeruginosa* clones through triparental mating as described above.

*P. aeruginosa* clones harboring the pSW-1 plasmid were selected on M9-citrate with 300 µg/mL carbenicillin. The resulting clones were screened by PCR with primer pairs 3, 4 to verify *hfq* deletion, which was confirmed by DNA sequencing of PCR products. Positive clones were grown in the absence of antibiotics to curate pSW-1 plasmid, and eventually carbenicillin-sensitive clones were selected. PCR primers used are listed in **Supplementary Table 1C**.

### 2.3 Minimum inhibitory concentration assay

MIC assays were performed with the microdilution method. Overnight cultures of *P. aeruginosa* strains grown in LB media were inoculated at OD_600_ of 0.1 and grown at 37 °C with shaking for 1.5 h in LB media, with the addition of 0.2% arabinose and, where needed, other supplements. Bacteria were centrifuged and resuspended in fresh media, then 5x10^4^ CFU were inoculated in 100 µL in a 96-well plate, containing 2-fold serial dilutions of meropenem, in LB with the necessary supplements. The plates were incubated at 37 °C with shaking in a Sunrise absorbance microplate reader (TECAN), where the OD_600_ was monitored every 14 minutes for 20 h. The growth data generated were analyzed to generate the growth curve, the endpoint OD_600_, the t_0_-t_5_ log-phase duration, and the area under the curve (AUC). Growth curves were generated by subtracting the blank value from the raw OD_600_ at each time point; OD_600_ values were plotted in a semi-log graph to generate the growth curves. Endpoint OD_600_ represents the OD_600_ reached at the end of the 20 h growth curve. The t_0_-t_5_ log phase duration was calculated by considering the time span in which the OD_600_ followed an exponential growth, in the first 5h of growth. For each growth curve, the OD_600_ values were transformed into natural logarithmic values ln(x), and a non-linear regression model was applied to evaluate the curve’s fitting to a straight line. The presence of a log-phase was confirmed if at least 4 points in the curve could fit the straight-line model with an R^2^ higher than 0.9. In case at least one of the biological replicates did not show an exponential phase, the strain was not considered to have a clear exponential phase in that condition. The AUC was estimated with the trapezoidal rule, as the sum of the areas under the curve calculated for each time interval. Comparison between two growth curves in different conditions was performed by two-way ANOVA. This was used to evaluate the impact that growth conditions (antibiotic concentration, sugar presence) had on bacterial growth. Data was analyzed to quantify the percentage of the source of variability that could be attributed to time, growth condition, or a combination of the two variables.

### 2.4 Statistical analysis

All experiments were performed with at least three independent biological replicates; statistical analysis was performed using GraphPad Prism 6.

## 3 Results

### 3.1 The intracellular levels of c-di-GMP conditionate *P. aeruginosa* susceptibility and tolerance to meropenem

**Figure 1:**
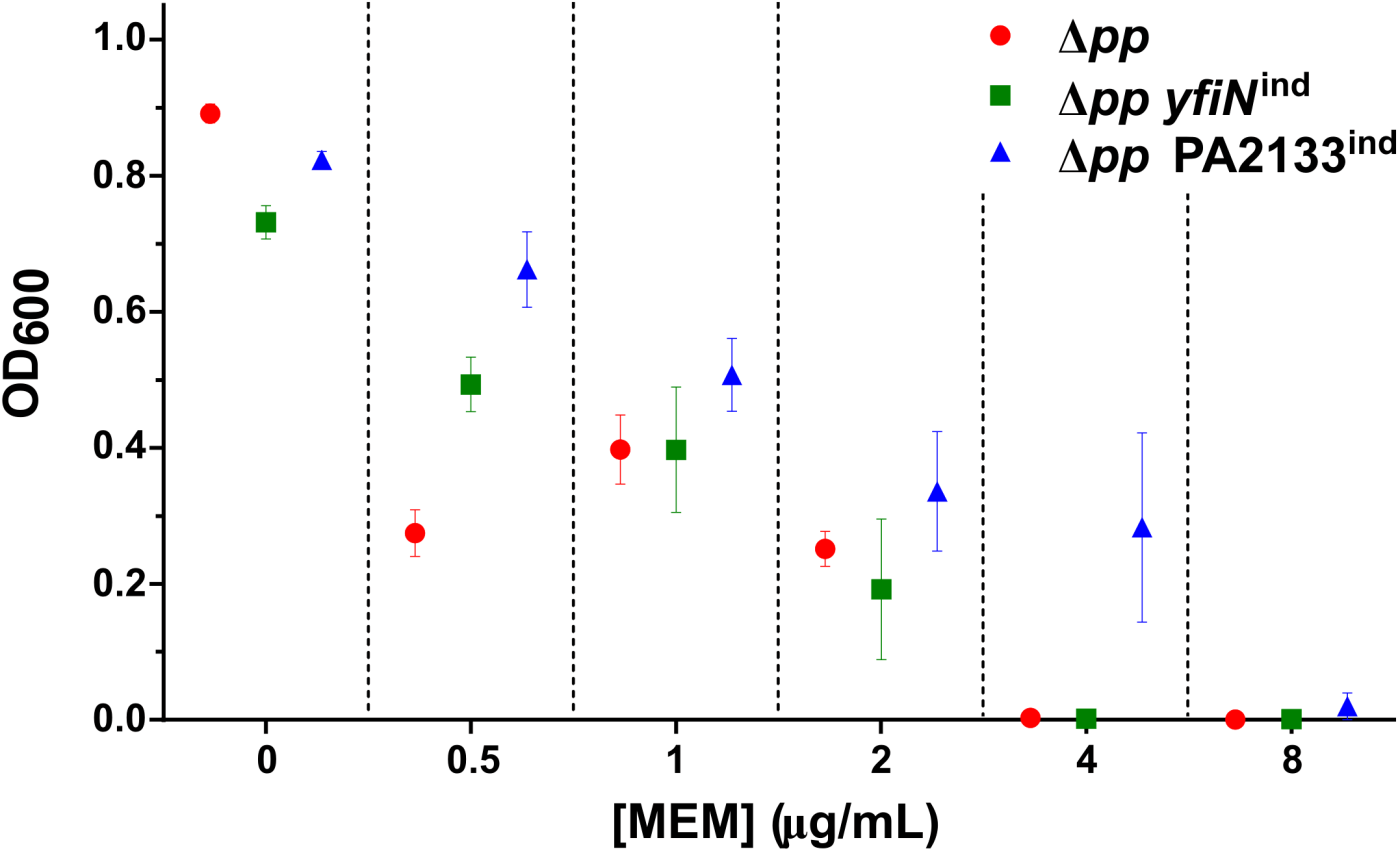
Influence of c-di-GMP levels on *P. aeruginosa* meropenem resistance. Endpoint OD_600_ reached by PAO1 Δ*pp*, PAO1 Δ*pp yfiN*^ind^, and PAO1 Δ*pp* PA2133^ind^, after 20 h growth at different meropenem (MEM) concentrations. Each dot represents the mean of three independent biological replicates; error bars represent the standard error of the mean.

**Figure 2:**
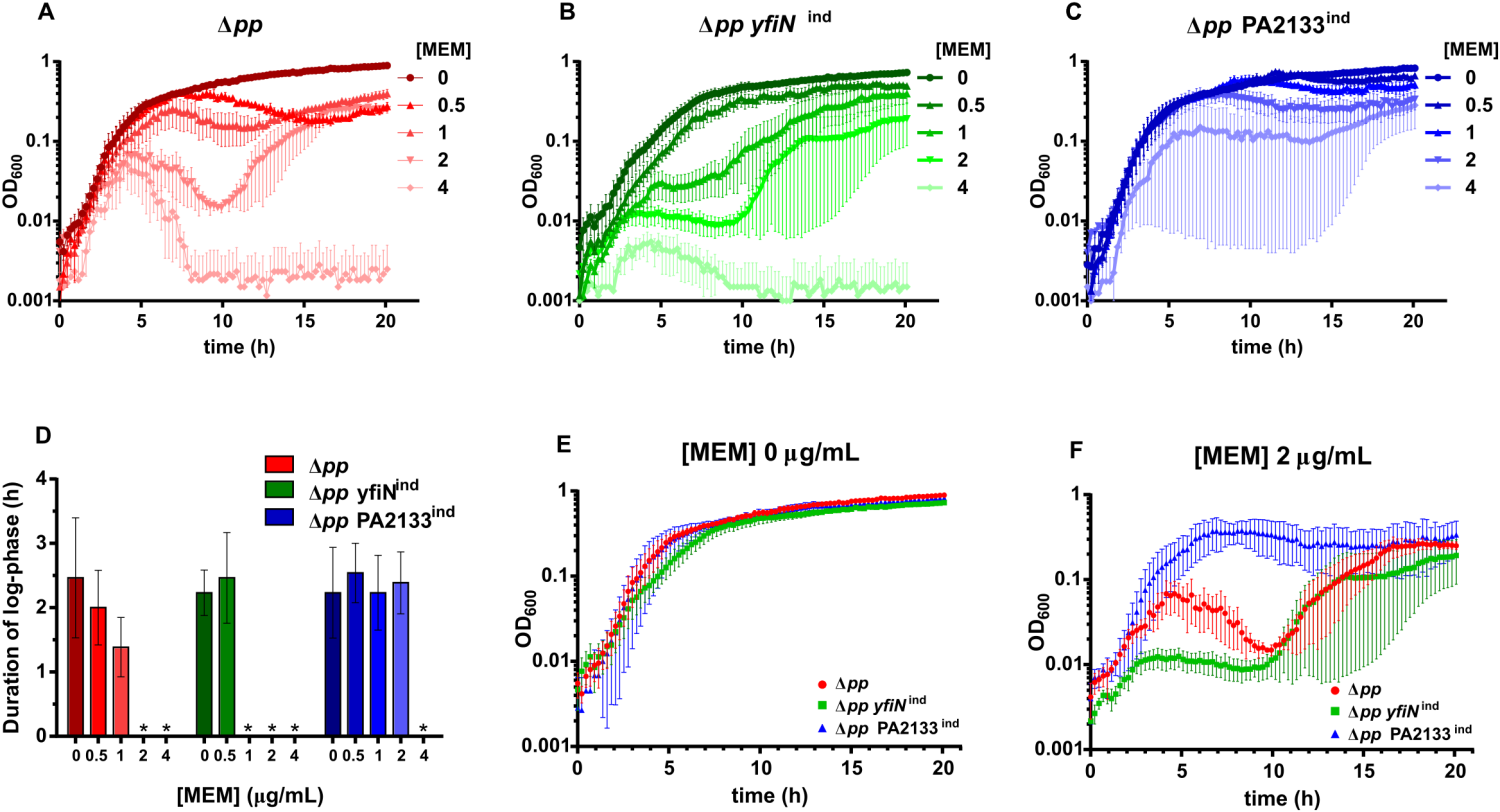
Influence of c-di-GMP levels on *P. aeruginosa* growth in the presence of sub-inhibitory concentration of meropenem. **(A–C)** Growth curves of PAO1 Δ*pp*, PAO1 Δ*pp yfiN*^ind^, and PAO1 Δ*pp* PA2133^ind^, respectively, in the presence of different concentrations of meropenem (MEM). Each dot represents the mean of three independent biological replicates; error bars represent the standard error of the mean. **(D)** Duration of the log-phase for each strain at different MEM concentrations. Log-phase was calculated for every single independent replicate, evaluating the number of points that could fit a straight line in a non-linear regression model with an R^2^ higher than 0.9. In case one or more growth curve replicates did not have a log-phase, the strain was considered not to have a clear log-phase at that MEM concentration. The absence of a clear log-phase is indicated with an asterisk. **(E)** Growth curves of PAO1 Δ*pp*, PAO1 Δ*pp yfiN*^ind^, and PAO1 Δ*pp* PA2133^ind^ in the absence of the antibiotic. **(F)** Growth curves of PAO1 Δ*pp*, PAO1 Δ*pp yfiN*^ind^, and PAO1 Δ*pp* PA2133^ind^ at 2 µg/mL MEM. Each dot represents the mean of three independent biological replicates; error bars represent the standard error of the mean.

We investigated whether intracellular c-di-GMP levels affect *P. aeruginosa* susceptibility to meropenem and tolerance at sub-inhibitory concentrations. To this end, we used the strains PAO1 Δ*pp yfiN*^ind^ and PAO1 Δ*pp* PA2133^ind^, PAO1 derivatives with altered c-di-GMP levels, which express the diguanylate cyclase YfiN or the phosphodiesterase PA2133, respectively, under the arabinose-inducible *P_BAD_* promoter (Pawar et al., 2016; Leoni et al., 2017). Compared to the parental PAO1 Δ*pp* strain—modified from PAO1 to prevent cell clumping and pellicle formation at high c-di-GMP levels by deleting the first four genes of the *pel* and *psl* operons—these strains exhibit elevated or reduced c-di-GMP levels, respectively. Meropenem susceptibility was assessed using standard serial dilution assays to determine the minimum inhibitory concentration (MIC). The MIC was 4 µg/mL for PAO1 Δ*pp* and PAO1 Δ*pp yfiN*^ind^, whereas PAO1 Δ*pp* PA2133^ind^ displayed increased resistance with an MIC of 8 µg/mL (**Figure 1**). To evaluate tolerance to sub-inhibitory meropenem concentrations, growth was monitored over 20 hours at increasing antibiotic concentrations (up to 4 µg/mL) (**Figures 2A–C**). At 0.5 and 1 µg/mL meropenem, the parental PAO1 Δ*pp* strain maintained an initial logarithmic growth phase (log-phase) comparable to that without antibiotic, with only a shortened duration (**Figure 2D**). At 2 and 4 µg/mL, the bacteriolytic effect of meropenem became evident, as growth curves deviated significantly from the typical exponential pattern (**Figure 2D**). In PAO1 Δ*pp yfiN*^ind^, initial growth began to deviate from the exponential pattern at 1 µg/mL meropenem (**Figure 2D**), indicating lower tolerance than the parental strain. Furthermore, after this initial growth phase, the OD_600_ values reached by PAO1 Δ*pp yfiN*^ind^ at 1, 2, and 4 µg/mL meropenem were lower than those of PAO1 Δ*pp* under the same conditions. Conversely, PAO1 Δ*pp* PA2133^ind^ showed altered log-phase only at 4 µg/mL **Figure 2D**, revealing significantly higher tolerance to bacteriolytic effects of meropenem.

Growth curve analysis also showed strain-specific meropenem response profiles extending beyond the log-phase, encompassing the onset and progression of the stationary phase, indicating that intracellular c-di-GMP levels influence not only exponential growth but also long-term survival dynamics under antibiotic stress. In PAO1 Δ*pp* (**Figure 2A**), between 2.5 and 10 hours after inoculation—an interval spanning the onset of the stationary phase and early stationary phase without antibiotics—increasing meropenem concentrations progressively accelerated lysis cell death kinetics as reflected by the increasingly rapid decline in optical density (OD_600_). At MIC (4 µg/mL), meropenem induced extensive cell lysis, reducing OD_600_ to baseline levels. Unexpectedly, at 1 and 2 µg/mL meropenem, PAO1 Δ*pp* entered a secondary growth phase during the 10–20 hour monitoring period, corresponding to the late stationary phase in antibiotic-free conditions.

Different from PAO1 Δ*pp*, PAO1 Δ*pp yfiN*^ind^ exhibited a growth plateau at 1 and 2 µg/mL meropenem (**Figure 2B**) during the 2.5–10 hour interval. However, at MIC (4 µg/mL), meropenem induced extensive cell lysis, reducing OD_600_ to baseline levels. Similar to PAO1 Δ*pp*, PAO1 PAO1 Δ*pp yfiN*^ind^ showed a secondary growth phase after 10 hours at 1 and 2 µg/mL meropenem.

Between 2.5 and 20 hours, the scenario was completely different for PAO1 Δ*pp* PA2133^ind^. Meropenem concentrations up to 2 µg/mL did not perturb the transition from exponential growth to the stationary phase or the establishment of the stationary-phase plateau. At 4 µg/mL, although the initial growth phase of PAO1 Δ*pp* PA2133^ind^ deviated from the typical exponential pattern (see above), overall growth dynamics remained comparable to those observed at lower meropenem concentrations. Notably, the stationary-phase plateau persisted across all antibiotic concentrations until the end of the monitoring period.

**Figures 2E, F** compare growth curves of the three strains at 0 and 2 µg/mL meropenem, highlighting how distinct c-di-GMP levels drive markedly different responses to sub-inhibitory meropenem concentrations. The low c-di-GMP strain PAO1 Δ*pp* PA2133^ind^ exhibited the highest tolerance across all growth phases. In contrast, the high c-di-GMP strain PAO1 Δ*pp yfiN*^ind^ showed markedly reduced tolerance during the log-phase, while the parental PAO1 Δ*pp* displayed intermediate response. However, PAO1 Δ*pp* becomes highly susceptible to meropenem-induced lysis during the transition to stationary phase, a vulnerability greater than that of PAO1 Δ*pp yfiN*^ind^, likely due to slower cell division in the latter. Remarkably, both PAO1 Δ*pp* and PAO1 Δ*pp yfiN*^ind^ underwent secondary growth phase, ultimately reaching cell densities comparable to PAO1 Δ*pp* PA2133^ind^.

Collectively, these data demonstrate that reduced intracellular c-di-GMP enhances *P. aeruginosa* fitness under meropenem exposure. Among the tested strains, PAO1 Δ*pp* PA2133^ind^ exhibited the lowest susceptibility (MIC), and the highest tolerance, as also reflected by consistently larger area under the growth-curve—a measure of the total amount of growth over time—and diminished meropenem-induced variability of growth-curve across all sub-inhibitory concentrations (**Supplementary Figures 1A, B**).

### 3.2 c-di-GMP–mediated modulation of meropenem susceptibility and tolerance occurs independently of Hfq

**Figure 3:**
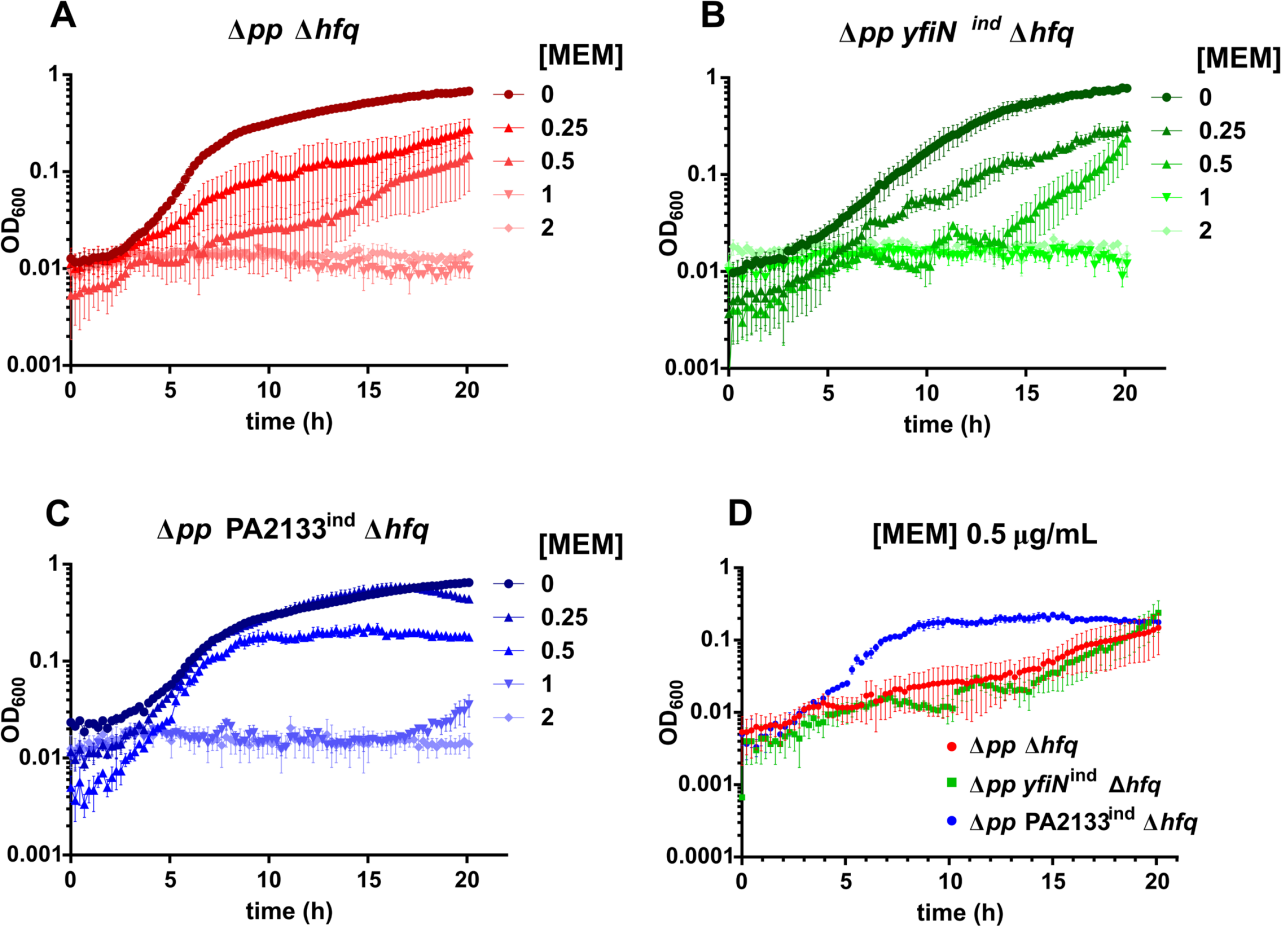
Influence of c-di-GMP levels and Hfq on *P. aeruginosa* growth in the presence of meropenem. **(A–C)** Growth curves of PAO1 Δ*pp* Δ*hfq*, PAO1 Δ*pp yfiN*^ind^ Δ*hfq*, and PAO1 Δ*pp* PA2133^ind^ Δ*hfq*, at different concentrations of meropenem (MEM). **(D)** Growth curves of PAO1 Δ*pp* Δ*hfq*, PAO1 Δ*pp yfiN*^ind^ Δ*hfq*, and PAO1 Δ*pp* PA2133^ind^ Δ*hfq* in the presence of 0.5 µg/mL MEM. Each dot represents the mean of three independent biological replicates; error bars represent the standard error of the mean.

We examined whether c-di-GMP–mediated modulation of meropenem susceptibility and tolerance involves the global regulator Hfq, which controls translation of the carbapenem-entry porins OprD and OpdP (Tamber and Hancock, 2006; Sonnleitner et al., 2020). To address this, we constructed *hfq* deletion mutants of PAO1 Δ*pp*, PAO1 Δ*pp yfiN*^ind^ and PAO1 Δ*pp* PA2133^ind^ and evaluated their meropenem MIC and tolerance profiles using the same assays described above. Comparative analysis revealed that the absence of Hfq did not abolish the differential meropenem response associated with c-di-GMP variation. Deletion of Hfq decreased MICs in all backgrounds, but the low c-di-GMP strain PAO1 Δ*pp* PA2133^ind^ Δ*hfq* remained comparatively more resistant, with an MIC of 2 µg/mL versus 1 µg/mL in the other mutants (**Supplementary Figure 2**). In addition, growth profiles at 0.25 and 0.5 µg/mL meropenem show that PAO1 Δ*pp* PA2133^ind^ Δ*hfq* exhibits the greatest tolerance and reduced variability in growth curves under antibiotic stress (**Figures 3A–D**; **Supplementary Figure S3**).

These results strongly suggest that c-di-GMP signaling influences meropenem susceptibility and tolerance through pathways that remain functional without Hfq, indicating that its modulatory effect operates independently of Hfq-mediated porin regulation.

### 3.3 Relative impact of c-di-GMP and carbon catabolite repression in modulating *P. aeruginosa* response to meropenem

**Figure 4:**
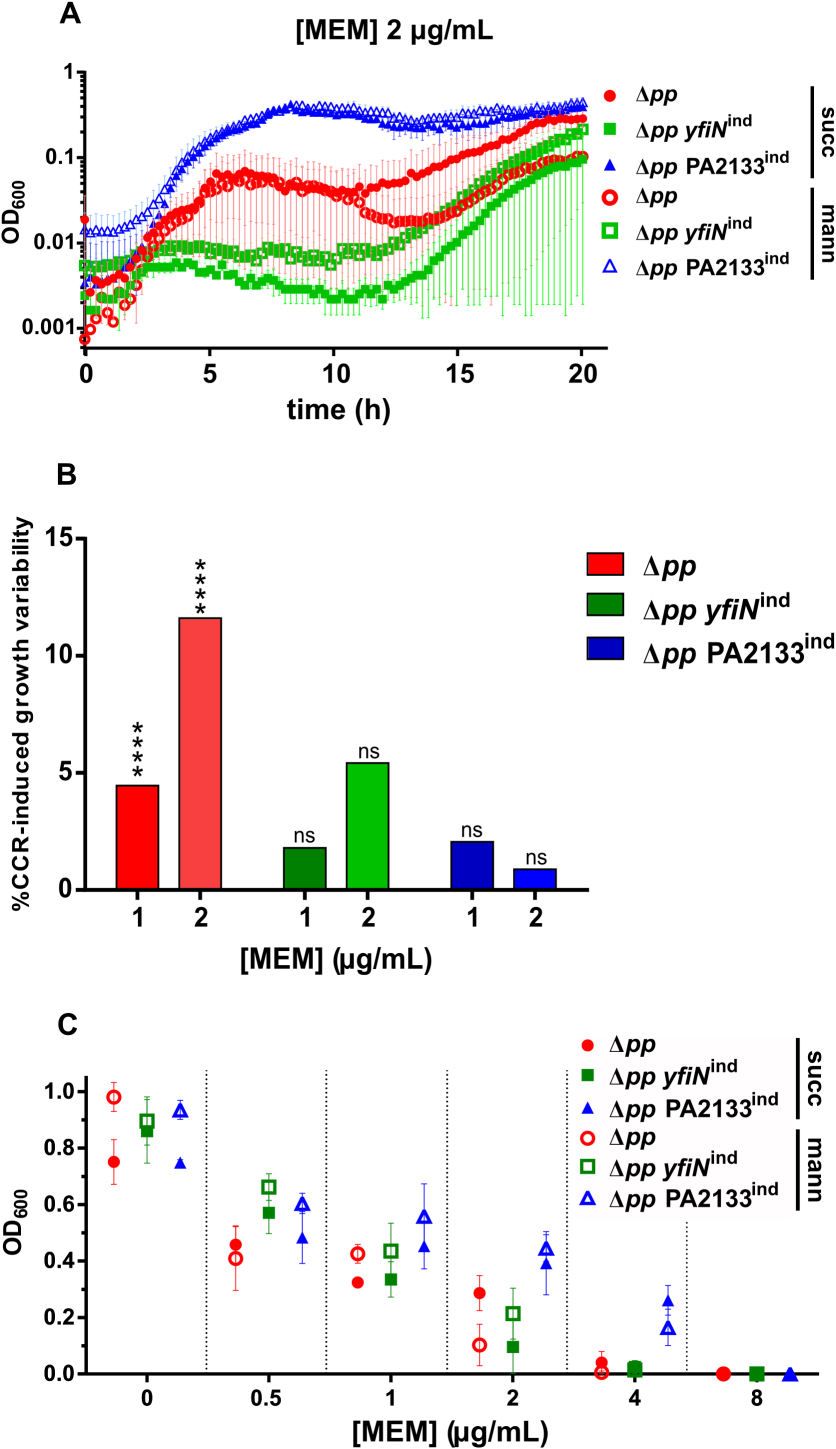
Influence of c-di-GMP levels and CCR on *P. aeruginosa* meropenem tolerance. **(A)** Growth curves of PAO1 Δ*pp*, PAO1 Δ*pp yfiN*^ind^, and PAO1 Δ*pp* PA2133^ind^ at 2 µg/mL meropenem (MEM), in the presence of 8 mM mannitol or succinate. Each dot represents the mean of three independent biological replicates; error bars represent the standard error of the mean. **(B)** Percentage of variability due to the interaction of time and carbon source supplement on the growth curves of PAO1 Δ*pp*, PAO1 Δ*pp yfiN*^ind^, and PAO1 Δ*pp* PA2133^ind^, at 1 and 2 µg/mL MEM, resulted from a two-way ANOVA analysis. P value: ****< 0.0001. **(C)** Endpoint OD_600_ reached by PAO1 Δ*pp*, PAO1 Δ*pp yfiN*^ind^, and PAO1 Δ*pp* PA2133^ind^, after 20 h growth at different MEM concentrations, in the presence of 8 mM mannitol or succinate. Each dot represents the mean of three independent biological replicates; error bars represent the standard error of the mean.

The Hfq-mediated modulation of OprD and OpdP porins was shown to be metabolically regulated by carbon catabolite repression (CCR) (Pusic et al., 2018; Sonnleitner et al., 2020). Preferred substrates typically reduce susceptibility, whereas non-preferred substrates increase it. To assess the respective contributions of c-di-GMP signaling and CCR in shaping meropenem response at sub-inhibitory concentrations, we monitored growth dynamics of PAO1 Δ*pp*, PAO1 Δ*pp yfiN*^ind^ and PAO1 Δ*pp* PA2133^ind^ over a 20-hour period at increasing antibiotic concentrations in media supplemented with either succinate, a preferred carbon source, or mannitol, a non-preferred source. **Figure 4A** compares growth profiles of the three strains at 2 µg/mL meropenem in media containing succinate or mannitol. Carbon source influenced growth dynamics only in the parental strain PAO1 Δ*pp*, predominantly during the secondary growth phase. Under mannitol, the decline in optical density before this phase was prolonged, delaying its onset. In contrast, PAO1 Δ*pp yfiN*^ind^ and PAO1 Δ*pp* PA2133^ind^ displayed comparable growth patterns regardless of carbon source. Statistical analysis confirmed significant CCR-induced variability of the growth curve exclusively for PAO1 Δ*pp* (**Figure 4B**). These results indicate that, while CCR can partially modulate antibiotic response in the parental strain, its influence disappears at both high and low c-di-GMP levels. Finally, MIC values for the three strains were unchanged across carbon sources (**Figure 4C**) and matched those without supplementation (**Figure 1**).

## 4 Discussion

Adaptive antibiotic resistance and tolerance can arise from regulatory pathways that reprogram gene expression to enhance bacterial fitness. Environmental conditions can alter bacterial physiology, thereby modulating the pathways that determine survival under antibiotic exposure. In this study, we investigated the contribution of c-di-GMP signaling to the regulation of *P. aeruginosa* susceptibility and tolerance to meropenem, a frontline carbapenem used in clinical treatment.

Overall, our results show that reduced intracellular c−di−GMP levels markedly increase meropenem resistance—doubling the MIC—and enhance tolerance at sub−inhibitory concentrations, whereas elevated c−di−GMP has the opposite effect. Growth−dynamic analyses show that c−di−GMP status shapes the entire trajectory of the antibiotic response, influencing both the early exponential phase and the onset and progression of the stationary phase. In the parental PAO1 Δ*pp* strain, meropenem impaired exponential growth and perturbed the transition to the stationary phase, revealing a window of increased susceptibility to lysis. Subsequently, a secondary growth phase emerged, suggesting that a resilient subpopulation survived antibiotic stress and re−initiated proliferation following tolerance development. High intracellular c-di-GMP in PAO1 Δ*pp yfiN*^ind^ exacerbated meropenem impact during exponential growth without abolishing the potential for secondary growth. Conversely, low c-di-GMP in PAO1 Δ*pp* PA2133^ind^ conferred broad tolerance, leaving exponential growth and transition to the stationary phase largely unaffected.

Carbapenem resistance in *P. aeruginosa* is strongly linked to Hfq-mediated translational repression of the porins OprD and OpdP, a role highlighted by the increased susceptibility of *hfq*-deficient mutants (Pusic et al., 2018; Sonnleitner et al., 2020). We deleted the *hfq* gene in all three c-di-GMP backgrounds and observed reduced resistance and tolerance. However, despite the loss of Hfq, the differential meropenem response associated with high and low c-di-GMP levels persisted, mirroring trends observed in Hfq-proficient counterparts. These findings indicate that c-di-GMP exerts its regulatory effect through mechanisms distinct from Hfq-mediated porin control, underscoring the existence of coexisting regulatory pathways that shape antibiotic response. These results do not exclude the possibility of crosstalk between the two regulatory pathways. c-di-GMP signaling could also have an impact on Hfq-mediated regulation of porins, for example through modulation of its expression and/or that of CrcZ, a mechanism that would integrate c-di-GMP signaling into CCR-associated metabolic feedback circuits responsive to carbon source availability.

CCR modulates carbapenem response through Hfq-mediated repression of entry-porins, thereby coupling nutrient availability to antibiotic influx (Pusic et al., 2018; Sonnleitner et al., 2020). We found that CCR exerts measurable effects only under restricted physiological conditions, specifically at sub-inhibitory meropenem concentrations in the parental PAO1 Δ*pp* strain. In contrast, its impact is negligible when intracellular c-di-GMP levels are perturbed. This suggests that meropenem tolerance changes driven by c-di-GMP signaling outweigh the localized effects of CCR on outer membrane permeability.

The increased meropenem fitness observed at low c-di-GMP levels in PAO1 Δ*pp* PA2133^ind^ may involve upregulation of MexAB-OprM efflux pump. In clinical *P. aeruginosa* strains, adaptive meropenem resistance commonly arises through loss of OprD-mediated uptake, β-lactamase-driven drug inactivation and overproduction of efflux pumps, frequently MexAB-OprM due to mutations affecting the repressor proteins MexR, NalC, or NalD (Glen and Lamont, 2021). *In vitro*, meropenem initially selects for OprD inactivation. Once OprD is absent, it imposes additional pressure favoring MexAB-OprM efflux pump overexpression (Köhler et al., 1999). In this context, our hypothesis that c−di−GMP modulates MexAB−OprM is supported by the observation that, under low intracellular c-di-GMP, MexB and OprM abundance significantly increases in *P. aeruginosa* PAO1, indicating enhanced efflux capacity (Chua et al., 2013). The molecular basis of this effect remains unknown, but low c−di−GMP may induce physiological cues that influence the activity of the efflux−pump repressors MexR, NalC, or NalD, or intersect with their regulatory pathways through c−di−GMP–responsive effectors.

The *P. aeruginosa* biofilm development cycle includes several stages, ranging from initial adhesion and maturation to matrix cavity formation for cell dispersal and the transition of dispersed cells back to planktonic state, enabling colonization of new niches (Thi et al., 2020). Given the central role of the second messenger c−di−GMP in controlling biofilm cycle and associated physiological states, we used strains engineered to maintain defined intracellular c−di−GMP levels and unable to produce important biofilm matrix components (i.e., pel and psl exopolysaccharides). Using these matrix−deficient backgrounds enabled us to decouple biofilm−dependent contributions (e.g., matrix−driven limitations on antibiotic penetration) from the intrinsic regulatory influence of c−di−GMP on meropenem response. Thanks to this experimental setup, we indicate that elevated c−di−GMP levels typical of biofilm cells do not confer increased meropenem fitness. Any resistance observed in biofilms therefore likely originates from pathways unrelated to c−di−GMP–dependent physiological states.

The transition from detachment to planktonic growth represents a distinct stage in the biofilm life cycle. Freshly dispersed cells exhibit physiological traits that differ from both biofilm-associated and planktonic populations, and are characterized by low intracellular c-di-GMP levels (Chua et al., 2013, 2015). Using a strain locked in a low c−di−GMP state allowed us to model the physiology of freshly dispersed cells and demonstrate that this condition enhances meropenem resistance and tolerance. A previous report indicated that low intracellular c-di-GMP levels in dispersed cells activate resistance pathways by upregulating proteins that counter antimicrobial peptides, including colistin (Chua et al., 2013). This response has been interpreted as a preemptive defense strategy, enabling dispersed cells to withstand subsequent antibiotic stress encountered during planktonic growth (Chua et al., 2013). This challenged the common view that dispersed cells are more susceptible to antibiotics than biofilm-associated counterparts. Showing that low c-di-GMP enhances meropenem fitness reinforces the view that dispersed *P. aeruginosa* cells constitute a physiologically resilient state, extending this principle from antimicrobial peptides to another class of antibiotics.

Our results have several potential clinical implications. Because environmental cues differ between laboratory tests and the host, c−di−GMP levels—and therefore meropenem response—may not be reliably captured by MIC−based diagnostics.

Dispersed cells with low c-di-GMP may represent a transient but clinically important population that is more tolerant to meropenem, increasing the risk of treatment failure or post−therapy regrowth. Besides, the increased tolerance associated with low c−di−GMP levels may enable prolonged survival under antimicrobial pressure, thereby facilitating the evolutionary progression toward stable meropenem resistance.

Finally, the use of biofilm-dispersing agents in combination with antibiotics is gaining attraction as an approach to improve antimicrobial efficacy (Hawas et al., 2022). Although effective for biofilm eradication, these strategies may produce dispersal cells with enhanced antibiotic tolerance, thereby promoting the adverse effects outlined above. On the other hand, compounds raising c−di−GMP levels may sensitize bacteria to meropenem treatment, yet their clinical use would need to account for the associated promotion of biofilm formation.

## Data Availability

The raw data supporting the conclusions of this article will be made available by the authors, without undue reservation.
