# Supplementary material for: Low levels of the second messenger c-di-GMP enhance tolerance and resistance to meropenem in Pseudomonas aeruginosa
Source: Front Cell Infect Microbiol. 2026 Mar 9;16:1775945. doi: 10.3389/fcimb.2026.1775945 (PMC13006659; doi:10.3389/fcimb.2026.1775945)
Supplement: Supplementary file 1 [file DataSheet1.pdf]

Supplementary Material

1 Supplementary Figures and Tables

1.1 Supplementary Figures

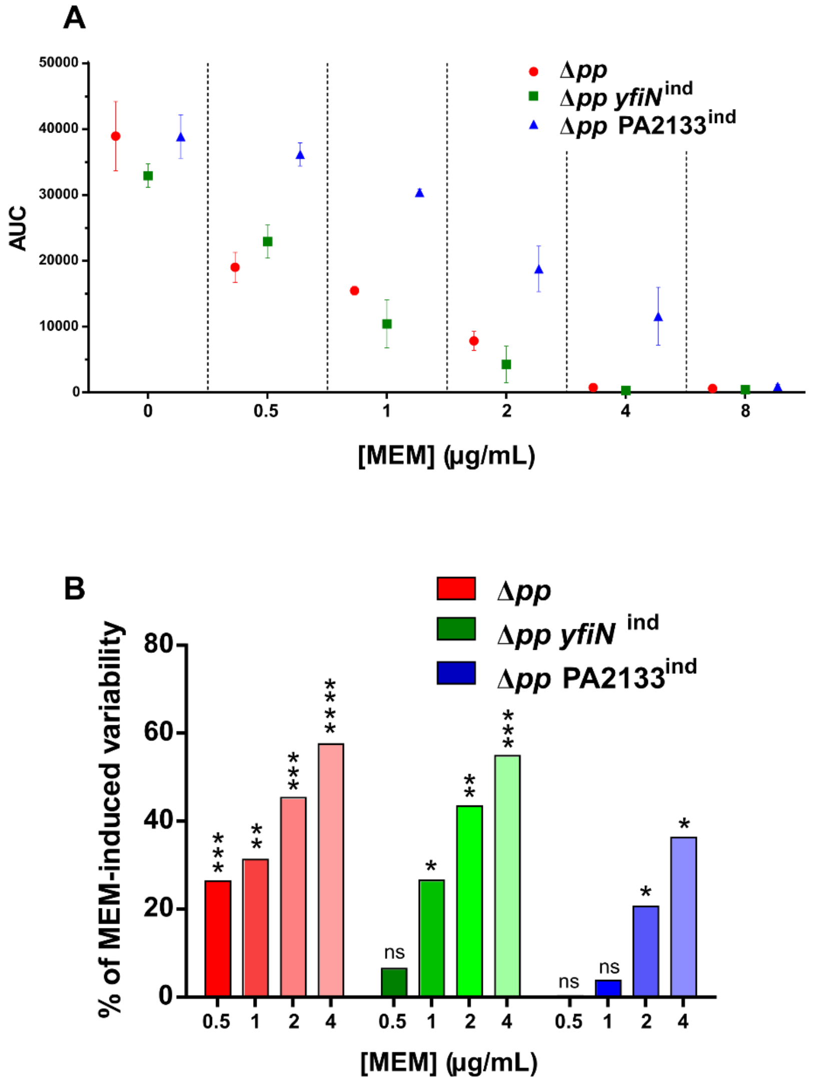

Figure S1. Impact of c-di-GMP levels on *P. aeruginosa* fitness in the presence of meropenem. (A) Area under the curve (AUC) values for PAO1 Δ*pp*, PAO1 Δ*pp yfiN*^{ind}, and PAO1 Δ*pp* PA2133^{ind} growth at different concentrations of meropenem (MEM). Each dot represents the mean of three independent biological replicates, expressed as AUC arbitrary units. Error bars represent the standard error of the mean. (B) Percentage of variability due to MEM concentrations on the growth curves of PAO1 Δ*pp*, PAO1 Δ*pp yfiN*^{ind}, and PAO1 Δ*pp* PA2133^{ind} resulted from a two-way ANOVA analysis, comparing the curve at the given MEM concentration with the growth in the absence of the antibiotic. P value is summarized with asterisks, $P < 0.05$ *, $P < 0.01$ **, $P <0.001$ ***, $P < 0.0001$ ****.

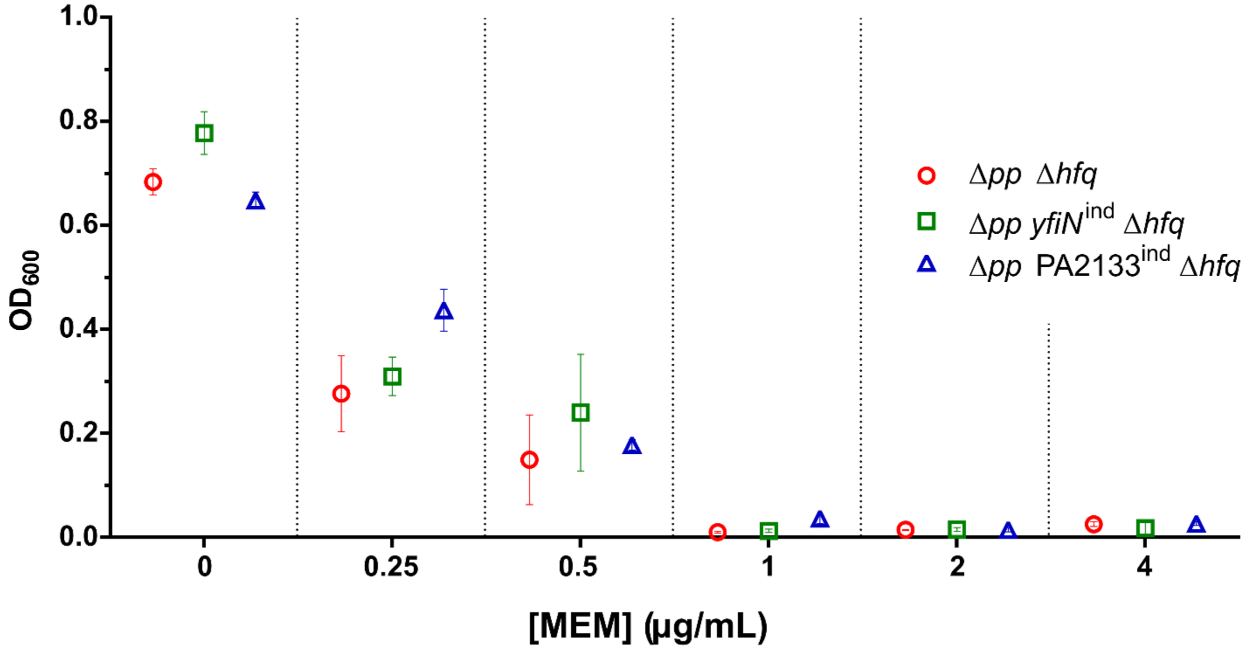

Figure S2. Influence of c-di-GMP levels on *P. aeruginosa* meropenem resistance in the absence of Hfq. Endpoint OD_{600} reached by PAO1 Δ*pp* Δ*hfq*, PAO1 Δ*pp yfiN*^{ind} Δ*hfq*, and PAO1 Δ*pp* PA2133^{ind} Δ*hfq* after 20 h growth at different meropenem (MEM) concentrations. Each dot represents the mean of three independent biological replicates; error bars represent the standard error of the mean.

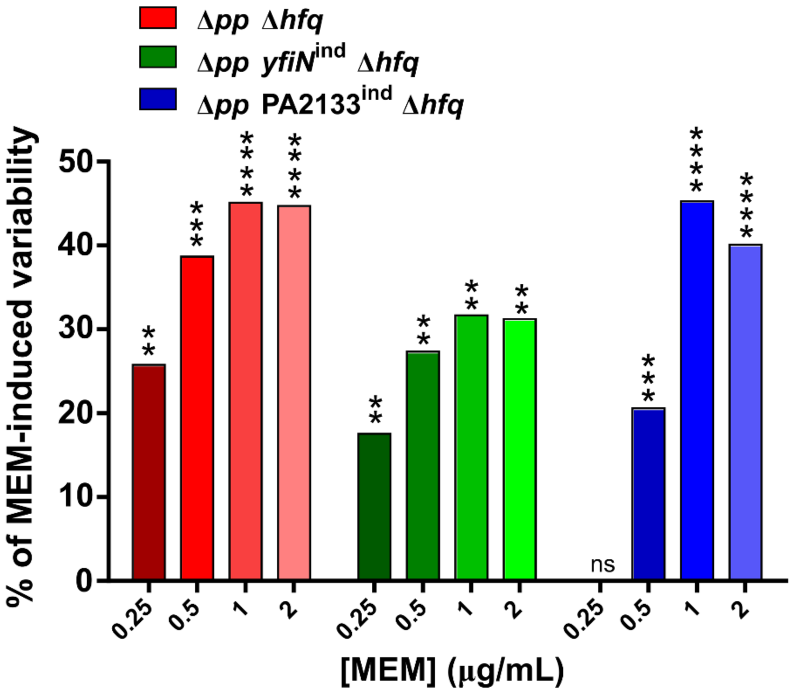

Figure S3. Effects of c-di-GMP on bacterial growth with meropenem in the absence of Hfq. Percentage of variability due to meropenem (MEM) concentrations on the growth curves of PAO1 Δ*pp* Δ*hfq*, PAO1 Δ*pp yfiN*^{ind} Δ*hfq*, and PAO1 Δ*pp* PA2133^{ind} Δ*hfq*, resulted from a two-way ANOVA analysis, comparing the curve at the given MEM concentration with the growth in the absence of the antibiotic. P value is summarized with asterisks, P < 0.01 **, P <0.001 ***, P < 0.0001 ****.

1.2 Supplementary Tables

Table S1A Bacterial strains

**Strain name**	**description**	**reference**
P. aeruginosa		
PAO1 Δ*pp*	PAO1 Δ*pelABCD* Δ*pslABCD*, (markerless)	(1)
PAO1 Δ*pp yfiN*^{ind}	PAO1 Δ*pelABCD* Δ*pslABCD::*P_{BAD} *-YfiN*^{ind} *(single-copy chromosomal integration),* Tc^R	(1)
PAO1 Δ*pp* PA2133^{ind}	PAO1 Δ*pelABCD*, Δ*pslABCD::*P_{BAD}*-PA2133*^{ind} *(single-copy chromosomal integration),* Tc^R	(1)
PAO1 Δ*pp* Δ*hfq*	Derivative of PAO1 Δ*pp,* Δ*hfq* (markerless)	This work
PAO1 Δ*pp yfiN*^{ind} Δ*hfq*	Derivative of PAO1 Δ*pp yfiN*^{ind}, Δ*hfq* (markerless)	This work
PAO1 Δ*pp* PA2133^{ind} Δ*hfq*	Derivative of PAO1 Δ*pp* PA2133^{ind}, Δ*hfq* (markerless)	This work
E. coli		
HB101(pRK600)	Helper strain for conjugative triparental mating	(2)

E. coli DH5α (pSW-1)	*E. coli* strain carrying the pSW-1 suicide plasmid, used for mutant generation.	(3)
E. coli CC118 λ*pir*	Δ(*ara-leu*), *araD*, Δ*lacX74*, *galE*, *galK*, *phoA*, *thi1*, *rpsE*, *rpoB*, *argE* (Am), *recA1*, lysogenic (λ*pir*)	(2)

Table S1B Plasmid used

**plasmid**	**description**	**reference**
pSEVAPA14-Δ*hfq*	pSEVA612S derivative, Gm^R	(4)
pSW-1	OriRK2, xylS, Pm-->I-SceI, Apr	(3)

Table S1C Oligonucleotides

**Oligo #**	**Oligo name**	**Sequence**	**Use**
1	pSEVA_f	TAAAACGACGGCCAGTATAG	Primers on pSEVA612S vector to verify the constructs with TS1 and TS2 and deletion mutants in combination with oligo 6
2	pSEVA_r	AGCTATGACCATGATTACG	
3	S1hfqPA14_Up_f	ATCCGCACCGCCTGATCG	primer annealing 380 bp upstream the *hfq* gene for deletion verification
4	TS2hfqPA14_Down_r	GGGTGGCGAACAACTGGT	primer annealing 307 bp downstream the *hfq* gene for deletion verification

References

1. Pawar SV, Messina M, Rinaldo S, Cutruzzolà F, Kaever V, Rampioni G, Leoni L. 2016. Novel genetic tools to tackle c-di-GMP-dependent signalling in Pseudomonas aeruginosa. J Appl Microbiol 120:205–217.

2. Kessler B, de Lorenzo V, Timmis KN. 1992. A general system to integrate lacZ fusions into the chromosomes of gram-negative eubacteria: regulation of the Pm promoter of the TOL plasmid studied with all controlling elements in monocopy. MGG Molecular & General Genetics 233:293–301.

3. Wong SM, Mekalanos JJ. 2000. Genetic footprinting with mariner-based transposition in Pseudomonas aeruginosa. Proc Natl Acad Sci U S A 97:10191–10196.

4. Carloni S, Macchi R, Sattin S, Ferrara S, Bertoni G. 2017. The small RNA ReaL: a novel regulatory element embedded in the Pseudomonas aeruginosa quorum sensing networks. Environ Microbiol 19:4220–4237.
